# Classic and targeted anti‐leukaemic agents interfere with the cholesterol biogenesis metagene in acute myeloid leukaemia: Therapeutic implications

**DOI:** 10.1111/jcmm.15339

**Published:** 2020-05-25

**Authors:** Fangli Chen, Xue Wu, Cristina Niculite, Marilena Gilca, Daniela Petrusca, Adriana Rogozea, Susan Rice, Bin Guo, Shawn Griffin, George A. Calin, H. Scott Boswell, Heiko Konig

**Affiliations:** ^1^ Melvin and Bren Simon Cancer Center Indiana University Indianapolis IN USA; ^2^ University of Medicine and Pharmacy ‘Carol Davila’ Bucharest Romania; ^3^ Division of Cancer Medicine Department of Experimental Therapeutics The University of Texas, MD Anderson Cancer Center Houston TX USA

**Keywords:** acute myeloid leukaemia, cholesterol, hypoxia, statins

## Abstract

Despite significant advances in deciphering the molecular landscape of acute myeloid leukaemia (AML), therapeutic outcomes of this haematological malignancy have only modestly improved over the past decades. Drug resistance and disease recurrence almost invariably occur, highlighting the need for a deeper understanding of these processes. While low O_2_ compartments, such as bone marrow (BM) niches, are well‐recognized hosts of drug‐resistant leukaemic cells, standard in vitro studies are routinely performed under supra‐physiologic (21% O_2_, ambient air) conditions, which limits clinical translatability. We hereby identify molecular pathways enriched in AML cells that survive acute challenges with classic or targeted therapeutic agents. Experiments took into account variations in O_2_ tension encountered by leukaemic cells in clinical settings. Integrated RNA and protein profiles revealed that lipid biosynthesis, and particularly the cholesterol biogenesis branch, is a particularly therapy‐induced vulnerability in AML cells under low O_2_ states. We also demonstrate that the impact of the cytotoxic agent cytarabine is selectively enhanced by a high‐potency statin. The cholesterol biosynthesis programme is amenable to additional translational opportunities within the expanding AML therapeutic landscape. Our findings support the further investigation of higher‐potency statin (eg rosuvastatin)–based combination therapies to enhance targeting residual AML cells that reside in low O_2_ environments.

## 1 INTRODUCTION

The dramatic progress in deciphering the molecular architecture of acute myeloid leukaemia (AML) has led to the development of a new generation of targeted therapeutic agents, such as the multitargeted kinase inhibitor midostaurin, the isocitrate dehydrogenase (IDH) inhibitors ivosidenib and enasidenib, and the B‐cell lymphoma (Bcl)‐2 and Hedgehog pathway inhibitors venetoclax and glasdegib, respectively, which recently received FDA approval.^1^, ^2^, ^3^, ^4^, ^5^ Despite these undeniable advances, only a limited subset of patients is expected to benefit from such agents, due to the vast molecular heterogeneity and complex clonal architecture of AML.^6^, ^7^ Based on extensive pre‐clinical and clinical literature, single anti‐AML drugs, whether targeted or not, fail to eliminate minimal residual disease (MRD), with relapse generally occurring within three years from diagnosis.^8^, ^9^, ^10^, ^11^, ^12^ Overall, the disconnect between the wealth of basic knowledge and dismal clinical outcome remains a defining feature of AML, with the vast majority of patients still relying on cytarabine, a nonspecific nucleoside analog introduced more than four decades ago, as the backbone of most chemotherapeutic regimens.^13^

Our efforts concentrate on the development of combinations based on existing AML agents by identifying and exploiting vulnerabilities associated with neoplastic microenvironmental hallmarks. While the impact of O_2_ abundance on cell physiology and drug responses has been extensively studied in relationship to solid tumours,^14^, ^15^ similar studies in the context of haematological malignancies are comparatively rare. Several lines of evidence suggest that O_2_‐deprived (hypoxic) niches within the bone marrow (BM) play a central role in AML drug resistance and disease relapse.^16^, ^17^ In tissue culture experiments, O_2_ concentrations between 1% and 5% are thought to better mimic oxygenation levels experienced by leukaemic cells in their natural environment.^18^, ^19^, ^20^, ^21^ Despite accumulating evidence for diverse artefact‐producing effects of excessive oxygenation,^22^, ^23^ AML in vitro studies are still routinely performed under atmospheric oxygenation (21% O_2_, ambient air), with potentially significant translational consequences.

In the present study, we investigated the effects of two clinically relevant agents, cytarabine and quizartinib, the former representing the classic anti‐AML cytotoxic drug, while the latter being representative for the new wave of highly specific kinase inhibitors,^24^ against AML cells under O_2_‐controlled conditions. Drug doses chosen for the proteo‐transcriptomic studies (cytarabine 1 µmol/L; quizartinib 1 nmol/L) represent a compromise between sublethal and clinical relevance: sufficient for a detectable expected response (eg apoptosis), but still compatible with a majority of cells remaining viability. Our working model is that the surviving pool exhibits shifts in molecular programmes that are critical for surviving the first therapeutic hit. Of note, we used a conceptually similar strategy to extract information about potential synergistic therapeutic partnerships in the context of solid tumours.^25^, ^26^ For practical reasons, the initial screens were performed in an established AML cell line (Molm14, M14), with subsequent validation experiments extended to a diverse panel, including primary leukaemic cells.

The results of our integrated screens converged towards a key role of lipid biosynthetic programmes, and in particular cholesterol biogenesis, during AML response to therapy. The results presented herein have significant translational implications, as they provide support for the value of adding higher‐potency statins (eg rosuvastatin) to classical and emerging AML therapies.

## 2 METHODS

### 2.1 Cell culture and reagents

Human AML cell lines, Molm14, and the relatively cytarabine‐resistant cell lines OCI‐AML3 and THP‐1^27^ (Figure S2) were obtained from the Deutsche Sammlung von Mikroorganismen und Zellkulturen. All cells were cultured in RPMI 1640 medium with 10% FBS and 100 U/mL streptomycin/penicillin in a humidified atmosphere at 37°C and 5% CO_2_. Quizartinib (AC220) and cytarabine were purchased from Selleck Chemicals, dissolved in dimethyl sulphoxide (DMSO) at stock concentrations of 10 mmol/L and stored at −80°C. Rosuvastatin was purchased from Tokyo Chemical Industry. Pravastatin, terbinafine, bempedoic acid and zoledronic acid were purchased from Selleck Chemicals. Unless otherwise indicated, all drugs were dissolved in DMSO at stock concentrations of 100 mmol/L and stored at −20°C. Fluorescein isothiocyanate‐Annexin V antibody and propidium iodide were purchased from BD Pharmingen and Sigma‐Aldrich.

### 2.2 Patient samples

Acute myeloid leukaemia blasts derived from whole blood or the bone marrow from 10 newly diagnosed (ND), previously untreated AML patients who consecutively presented to our institution were obtained under guidelines approved by the Institutional Review Board of the Indiana University (Indianapolis, IN, USA). All patients gave informed consent according to the Declaration of Helsinki. Blasts were separated by centrifugation over a layer of Ficoll‐Paque PLUS (GE Healthcare), collected as the buffy coat and then either stored in freezing medium (foetal bovine serum with 10% dimethyl sulphoxide) in liquid nitrogen or plated immediately for experiments. The clinical characteristics of the patients from whom samples were derived are listed in Table 1.

**Table 1:** Patient characteristics

Patient no.	Age (y)	Gender, male (M)/female (F)	Cytogenetics	Molecular alterations identified
1	60	M	46, XY	BCOR, FLT3/TKD, PHF6, RUNX1
2	24	F	47, XX, +8	FLT3/ITD
3	58	M	45, XY, del (5)	TET2, TP53, RUNX1, JAK2
4	85	M	46, XY	Molecular testing deferred by patient
5	65	M	46, XY, t(9;11)	ASXL1
6	56	F	46, XX	FLT3/ITD, ATRX, CCT6B, KMT2C (MLL3), NPM1, PTPN11, RAD21
7	36	F	46, XX, inv (12)	FLT3/ITD, NPM1
8	75	F	46, XX	FLT3/ITD, NPM1
9	78	M	47, XY, +8	None
10	25	M	46, XY	None

### 2.3 MTT cell viability assays

1 × 10^4^ Molm14, THP‐1 and OCI‐AML3 cells or 15 × 10^4^ fresh primary cells suspended in RPMI 1640 culture medium with 10% FBS were seeded in 96‐well plates. Cells were treated with either drug in single agent or combined mode under ambient air (21% O_2_, normoxia) or hypoxic conditions (1% O_2_) for 48 hours as outlined for each experiment. Cell viability was assessed using MTT (3‐(4,5‐dimethylthiazol‐2‐yl)‐2,5‐diphenyltetrazolium bromide; Roche) in accordance with the manufacturer's recommended protocols. Briefly, 20 µL MTT solution was added into each well and incubated at 37°C for 2 hours. Plates were read on at 490 nm with a plate reader. Growth inhibition percentage was calculated from the optical density ratio of treatment to DMSO control after subtraction of background under 21% O_2_ and 1% O_2_, respectively. All assays were performed in triplicate, and the results were obtained in at least three independent experiments as outlined for each experiment. Combination indexes (CIs) were determined using CompuSyn software.

### 2.4 Apoptosis assay

A total of 5 × 10^5^ leukaemia cells were seeded in 6‐well plates and incubated with cytarabine and/or rosuvastatin under 21% or 1% O_2_ conditions as outlined for each experiment. After 48 hours, cells were harvested from the plates, washed twice with cold PBS and then re‐suspended in 100 µL binding buffer. 5 µL FITC‐Annexin V and 10 µL propidium iodide stock solution (50 µg/mL) were added to each sample and incubated at room temperature in the dark for 15 minutes. Cells were analysed on a BD Accuri (BD Biosciences).

### 2.5 RNA isolation and quantitative PCR

Molm14 and THP‐1 cells were treated with cytarabine for 48 hours. Total RNA was extracted by RNeasy Plus Kit (Qiagen) according to the manufacturer's instructions. cDNA was synthesized with reverse transcription kit (Thermo Fisher Scientific) as per the manufacturer's protocols. The PCR primers used in the study were as follows:

- HMGCS1 (F): CTCTTGGGATGGACGGTATGC and HMGCS1 (R): GCTCCAACTCCACCTGTAGG;
- LSS (F): GACGACCGATTCACCAAGAGCA and LSS (R): AGACATGCTCCTGGAAGGCAGT;
- MSMO1 (F): GCTGCCTTTGATTTGTGGAACCT and MSMO1 (R): CTGCACAACCAAAGCATCTTGCC;
- SQLE (F): CTCCAAGTTCAGGAAAAGCCTGG and SQLE (R): GAGAACTGGACTCGGGTTAGCT;
- CD36 (F): CAGGTCAACCTATTGGTCAAGCC and CD36 (R): GCCTTCTCATCACCAATGGTCC
- RN18S1 (F): ACCCGTTGAACCCCATTCGTGA and RN18S1 (R): GCCTCACTAAACCATCCAATCGG

RN18S1 expression was used as a control for RNA loading. Quantitative PCRs were performed using PowerUp SYBR Green PCR Master Mix (Applied Biosystems;) on 7900HT Real‐Time PCR System.

### 2.6 Total cholesterol quantification

Cellular cholesterol was measured using Wako cholesterol assay kit (#439‐17501; WAKO). In brief, Molm14, THP‐1 or primary AML cells were incubated with cytarabine and rosuvastatin in single agent or combined mode under 21% O_2_ or 1% O_2_ for 48 hours. Leukaemia cells were washed twice with ice‐cold PBS and re‐suspended in 150 µL PBS. Cellular lipids were extracted using 750 µL chloroform:methanol (2:1) solution. After extraction for 30 minutes on a rocker, samples were centrifuged at 1252 *g* at 4°C for 10 minutes and the lower chloroform phase was collected and air‐dried in a fume hood until all liquid evaporated. The lipids were then re‐suspended in 10% Triton X‐100/isopropanol. Total cholesterol content was measured using Wako cholesterol assay kit according to the manufacturer's instructions.

### 2.7 RNAseq analysis

Total RNA was evaluated for its quantity and quality using Agilent Bioanalyzer 2100. For RNA quality, an RNA integrity (RIN) number of 7 or higher was required. About 500 ng of total RNA was used for cDNA library preparation, including mRNA purification/enrichment, RNA fragmentation, cDNA synthesis, ligation of index adaptors and amplification, following the TruSeq Stranded mRNA Sample Preparation Guide, RS‐122‐9004DOC, Part# 15031047 Rev. E (Illumina, Inc). Each resulting indexed library was quantified, and its quality was accessed by Qubit and Agilent Bioanalyzer, and multiple libraries were pooled in equal molarity. Five microlitres of 2 nmol/L pooled libraries per lane was then denatured, neutralized and applied to the cBot for flow cell deposition and cluster amplification, before loading to HiSeq 4000 for sequencing (Illumina, Inc).

### 2.8 Gene set enrichment analysis (GSEA)

Gene Set Enrichment Analysis was performed following GSEA User Guide (The Broad Institute). Briefly, each condition was considered as a group and gene list was ranked with GSEA default ranking metrics. Gene sets from Molecular Signature Database were used in the analysis to identify the pathways significantly enriched in each group. Gene sets were permutated 1000 times to obtain empirical FDR‐corrected *P*‐values.

### 2.9 Reverse protein array (RPPA)

In brief, cell lysate samples were serially diluted twofold for 5 dilutions (undiluted, 1:2, 1:4, 1:8 and 1:16) and arrayed on nitrocellulose‐coated slides in an 11 × 11 format to produce sample spots. Sample spots were then probed with antibodies by a tyramide‐based signal amplification approach and visualized by DAB colorimetric reaction to produce stained slides. Stained slides were scanned on a Huron TissueScope scanner to produce 16‐bit tiff images. Sample spots in tiff images were identified and their densities quantified by Array‐Pro Analyzer. Relative protein levels for each sample were determined by interpolating each dilution curve produced from the densities of the 5‐dilution sample spots using a ‘standard curve’ (SuperCurve) for each slide (antibody). All relative protein level data points were normalized for protein loading and transformed to linear values.

### 2.10 Western blot analysis

Cell lysates were prepared in RIPA buffer containing protease inhibitor cocktail (Thermo Fisher Scientific). Proteins were separated by SDS‐polyacrylamide gel electrophoresis (SDS‐PAGE) and then transferred onto PVDF membranes (Bio‐Rad). Primary antibodies used in this study included anti–stearoyl‐CoA desaturase (anti‐SCD) (sc‐58420) and anti–fatty acid synthase antibody (A‐5) (anti‐FASN) (sc‐55580; Santa Cruz), as well as anti‐β‐tubulin (sc‐5274; Santa Cruz). Horseradish peroxidase‐conjugated secondary antibodies (NA931V) were from GE Healthcare. Antibody detection was performed using ECL™ Western blotting reagent kit (RP2106) (GE Healthcare).

### 2.11 High‐performance liquid chromatography (HPLC)

Rosuvastatin and pravastatin were quantified per high‐performance liquid chromatography coupled with electrospray ionization tandem mass spectrometry. Briefly, rosuvastatin and pravastatin were quantified from media using simvastatin as the internal standard and HPLC‐MS/MS (ABSciex 4000). For the samples and standards, rosuvastatin, pravastatin and simvastatin were extracted from media by the addition of 0.1 mol/L citric acid buffer (pH = 3.0) followed by liquid‐liquid extraction using ethyl acetate. The analytes were separated by a gradient mobile phase (acetonitrile: 5 mmol/L ammonium acetate, pH = 3.5 with acetic acid) with a Zorbax 300SB‐C8 150 × 4.6 mm 5 µm column. The mass spectrometer utilized an electrospray ionization probe run in both positive and negative modes. The multiple reaction monitoring (MRM) Q1/Q3 (*m/z*) transitions in positive mode for rosuvastatin and simvastatin are 482.1/258.2 and 419.4/199.0, respectively. The MRM Q1/Q3 (*m/z*) transition in negative mode for pravastatin is 423.0/321.2. The lower limit of quantification for both rosuvastatin and pravastatin is 1 ng/mL using 200 μL media.

### 2.12 Statistical analysis

Statistical significance of differences was determined by Student's *t* test. Difference was considered statistically significant when *P*‐value was <.05. Data are presented as mean ± standard error of the mean (SEM) unless stated otherwise.

## 3 RESULTS

### 3.1 Exposure to cytarabine and quizartinib leads to coordinated down‐regulation of the cholesterol biosynthesis pathway

**Figure 1:**
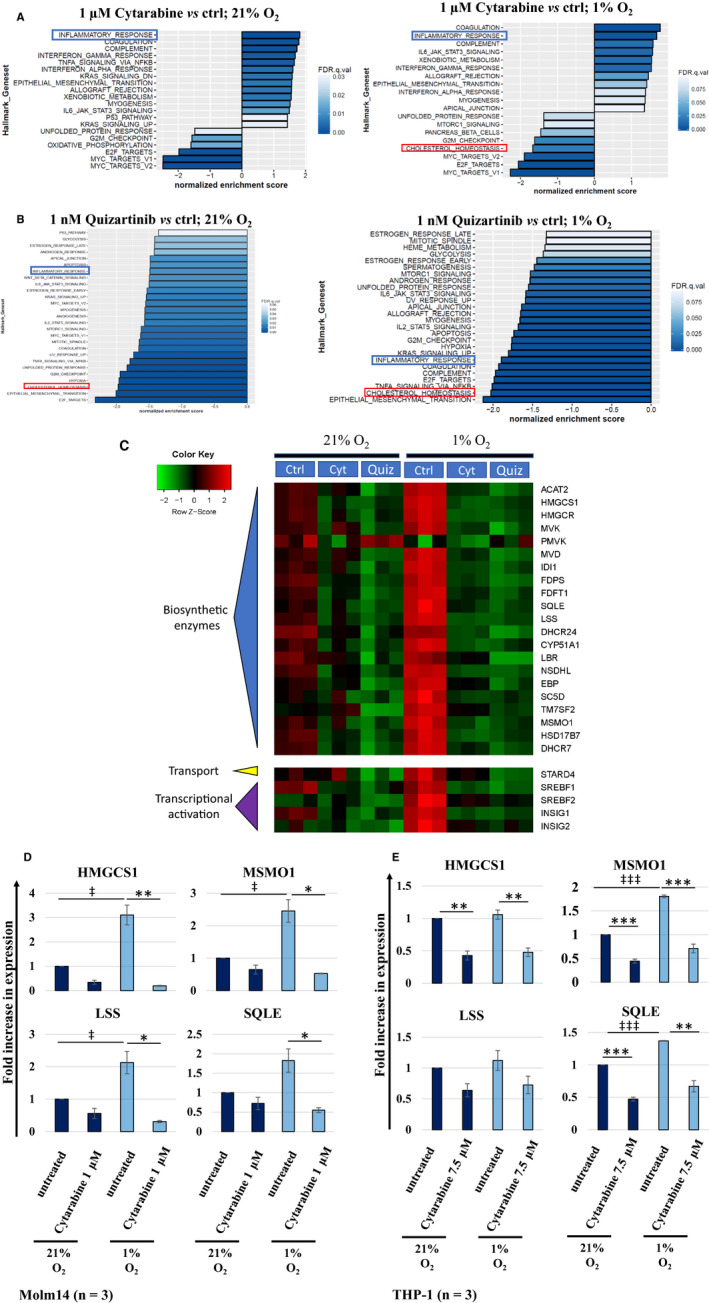
Coordinated downregulation of the cholesterol biosynthesis program by cytarabine and quizartinib. GSEA‐enrichment bar plots depicting the normalized enrichment scores of the most negatively and positively enriched gene sets in Molm14 cells exposed to (A) 1 μmol/L cytarabine and (B) 1 nmol/L quizartinib for 48 h under 21% and 1% O_2_ conditions. Among the differentially expressed genes, suppression of cholesterol biosynthesis was the cardinal feature of cytarabine and quizartinib responses under 1% O_2_. (C) RNA seq analysis revealed downregulation of the cholesterol biosynthesis program that is coordinately induced by treatment with cytarabine and quizartinib. The impact is enhanced under lower O_2_ tension. (Ctrl = untreated control; Cyt = cytarabine; Quiz = quizartinib). Focused quantitative RT‐PCR assays for specific members of the cholesterol biosynthetic program (HMGCS1, MSMO1, LSS and SQLE) confirmed the downregulatory effects of Cytarabine in (D) Molm14 and (E) THP‐1 cells. The negative effects of cytarabine treatment appears magnified under O_2_ deprived conditions. Higher doses of cytarabine were used for the relatively cytarabine resistant THP‐1 cells. Results represent mean ± SEM fold increase in mRNA expression versus the untreated control at 21% O_2_ (n = 3). Statistically significant differences in mRNA expression are indicated as follows: * or ^‡^ *P* < .05; ** or ^‡‡^ *P* < .01; *** or ^‡‡‡^ *P* < .001

We employed a transcriptome‐proteome profiling strategy based on RNAseq and RPPA for a preliminary portrait of the global impact of cytarabine and quizartinib on Molm14 cells. The former drug was chosen as it remains the staple of AML therapy,^13^ while the latter is a member of the new class of highly selective targeted agents transitioning from bench to bedside.^24^ Our premise was that an in vitro pharmacological treatment is particularly informative if a drug is used at a concentration that leaves the majority of cells viable, but sufficient to elicit molecular signatures consistent with its known mechanism of action. RNA and protein were extracted from Molm14 cells treated with either drug or corresponding controls, under O_2_‐replete or O_2_‐depleted conditions. Differentially expressed transcripts were determined by RNAseq analysis, followed by Gene Set Enrichment Analysis (GSEA) to identify the main pathways/programmes affected by drug treatment. Using the hallmark collections, GSEA identified ‘cholesterol homeostasis’ down‐regulation as a major common feature of cytarabine and quizartinib responses under 1% O_2_, while at 21% O_2_, the suppression hallmark passed the significance threshold in quizartinib sets only, and upon further inspection revealed a similar response, albeit less robust, following cytarabine. This coordinated shift following anti‐AML agents under high and low O_2_ is captured by the heatmaps shown in Figure 1A‐C and Figure S1A. There is a striking dominance of cholesterol biogenesis programme among the top 20 differentially expressed transcripts (or over 10 000 identified) under low O_2_ in response to either drug (see *P*‐value/FDR values in Tables S1A and S1B).

### 3.2 Confirmation of OMICS by quantitative RT‐PCR

RNAseq results were confirmed by quantitative RT‐PCR for several pathway components: 3‐hydroxy‐3‐methylglutaryl‐CoA synthase 1 (HMGCS1), methylsterol monooxygenase 1 (MSMO1), lanosterol synthase (LSS) and squalene monooxygenase (SQLE). As shown in Figure 1D, the transcripts were induced under 1% O_2_ and down‐regulated in response to cytarabine under both high and low oxygenation. Importantly, as depicted in Figure 1E, these responses were replicated in THP‐1 cells (and thus appear to occur irrespective of FLT3 mutational status). As suggested by RNAseq data, and confirmed by QRT‐PCR for specific members, the negative effect of drug treatment on this transcriptional programme appears magnified under hypoxic conditions. Transcriptional profiles revealed a striking sensitivity of cholesterol biosynthesis metagene to O_2_ deprivation, at least in Molm14 cells. While functional connections of this pathway to hypoxia are increasingly appreciated,^28^, ^29^, ^30^, ^31^ virtually all knowledge has been generated in the context of solid tumours, with little specific information derived from haematological malignancies.

### 3.3 Protein programme responses to anti‐AML agents

**Figure 2:**
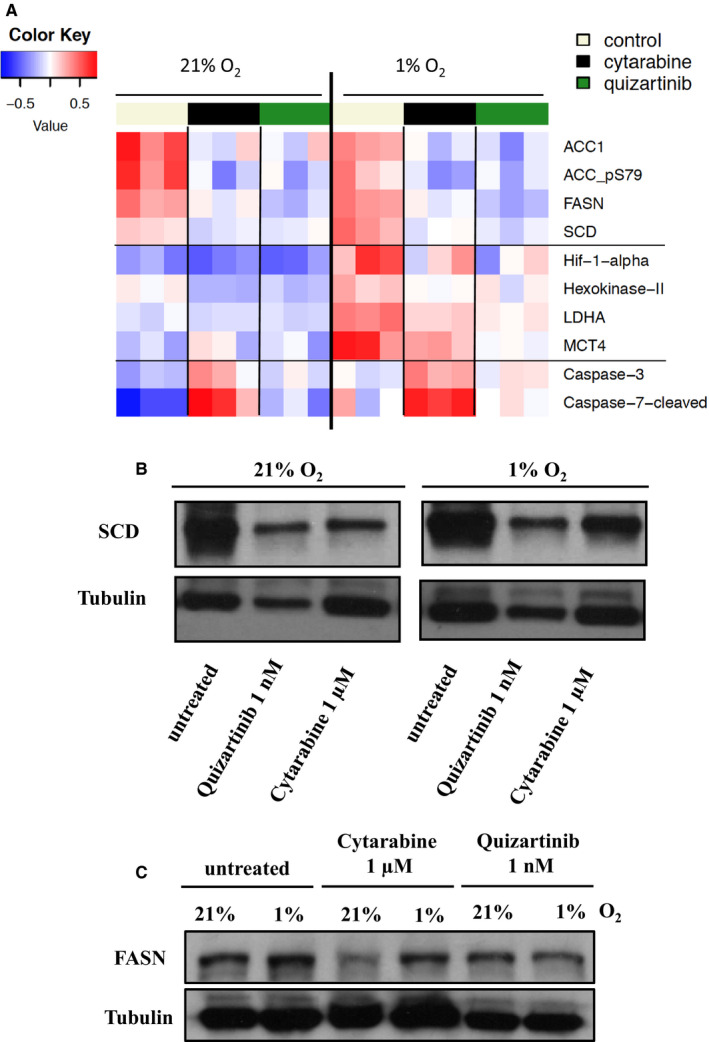
Protein programme responses to anti‐AML agents. A, In line with the data obtained from RNAseq experiments, RPPA‐based protein profiling demonstrates down‐regulation of rate‐limiting components of fatty acid biogenesis (FASN, SCD and acetyl‐CoA carboxylase), a pathway closely coordinated with cholesterol biosynthesis, in response to cytarabine and quizartinib. Hypoxia‐induced activation of HIF‐1‐alpha and several glycolytic programme effectors (hexokinase‐II, LDHA and MCT4) was stalled by cytarabine and quizartinib. In addition, cytarabine, but not quizartinib, displayed DNA damage and apoptosis‐inducing responses (Caspase‐7‐cleaved, Caspase‐3). B, Confirmation of RPPA results by Western blotting for SCD. C, Confirmation of RPPA results by Western blotting for FASN

A complementary dimension to the transcriptional portrait of drug response was provided by RPPA‐based comparisons (Figure 2A). RPPA is an antibody‐based quantitative assay that quantifies hundreds of cancer‐relevant proteins (total and phosphorylated), including critical components for survival and growth of AML cells. Most proteins and phosphoproteins undergo less than 10% change, suggesting that the system remains largely viable and interpretable. Cytarabine, but not quizartinib, displayed a visible DNA damage response and pro‐apoptotic footprint (cleaved Caspase‐7 and Caspase‐3). However, quizartinib treatment predominantly impacted cell cycle regulators (Table S1B). Activation of the hypoxia‐inducible factor 1 alpha (HIF‐1‐alpha)–driven programme and particularly its glycolytic metabolic arm appeared antagonized by cytarabine and quizartinib, at both RNA and protein data sets. Although RPPA in the current configuration does not include proteins primarily involved in cholesterol biogenesis, it yielded potentially significant clues regarding AML response programmes to the drugs, and the specific footprint of hypoxia. We found that a major biosynthetic pathway closely coordinated with cholesterol biosynthesis was down‐regulated following both treatments. This is consistent with the RNAseq data summarized above. Multiple rate‐limiting components of fatty acid biogenesis, including fatty acid synthase (FASN), stearoyl‐CoA desaturase‐1 (SCD) and acetyl‐CoA carboxylase (ACC1), were among the top suppressed proteins (with the caveat that functional suppressive serine 79–phosphorylated form of the latter was similarly decreased). RPPA results were confirmed by Western blotting for SCD and FASN (Figure 2B,C).

There are notable similarities, particularly under low O_2_, between cytarabine‐residual Molm14 cells and patient‐derived blasts that have become resistant to this agent in a xenograft AML model.^32^ The transcriptomic analysis by Farge and colleagues identified cholesterol biosynthesis as the top metagene down‐regulated in residual cells. Their supplemental data (table S3 in Ref.^32^) further revealed that virtually all pathway components present in our Figures 1C and 2A are among the highly significant depleted transcripts. The similarities extend to down‐regulation of the closely connected fatty acid biosynthesis programme (FASN, SCD).

### 3.4 Assessment of intracellular cholesterol levels in AML cell lines and primary cells after treatment with cytarabine in vitro

**Figure 3:**
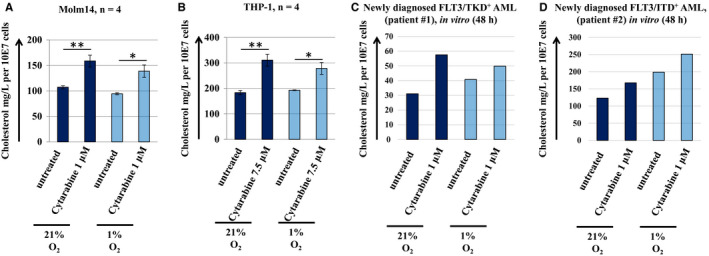
Assessment of intracellular cholesterol levels in AML cell lines and primary cells after treatment with cytarabine in vitro. Intracellular cholesterol levels increase in response to cytarabine under normoxic and hypoxic conditions in (A) Molm 14, (B) THP‐1 and primary cells derived from (C) FLT3/TKD and (D) FLT3/ITD mutated patients. Cells were incubated with 1 μmol/L cytarabine under 21% or 1% O_2_ for 48 h. Results represent mean ± SEM mg/L of cholesterol content per 107 cells ([Molm14, THP‐1], n = 4). Statistically significant differences in intracellular cholesterol content are indicated as follows: *P < .05; **P < .01

Appelbaum, Penn and colleagues noted that high mevalonate pathway activity and cholesterol abundance are hallmarks of AML.^33^, ^34^ Furthermore, most AML cells respond to cytotoxic agents, and in particular to cytarabine, by further increasing their total cholesterol content.^35^ In contrast, very limited, if any, quantitative information is currently available regarding the individual components of this complex pathway. Our work suggests that cytarabine treatment results in increased cholesterol levels in AML cell line and primary cell assays (Figure 3A‐D). The differences in cytarabine doses are generally consistent with CCLE drug sensitivity data sets (see Figure S2 for a summary of established AML cell lines). A similar response was further demonstrated on leukaemic cells assayed for cholesterol content immediately before and 24 hours after standard ‘7 + 3’ induction chemotherapy (Figure S3A,B). Overall, an increase in total cholesterol by cytarabine treatment was detected under both 1% and 21% O_2_ conditions in the in vitro studies.

Our data collectively suggest that cytarabine (and potentially quizartinib)‐residual cells ‘dial down’ energy‐intensive biosynthetic programmes, in particular biosynthesis of sterols and fatty acids, and switch to ‘scavenger’ mode. A central component of this switch may be up‐regulation of multifunctional translocase CD36/fatty acid translocase (FAT), as suggested by RNAseq profiles and independently confirmed by TaqMan quantitative RT‐PCR in Molm14 cells (Figure S1A‐C). This is again reminiscent of the differences between the residual versus original population in the recent in vivo study by Farge et al.^32^

### 3.5 Translational applications of cholesterol biosynthesis blockade using a potent HMG‐CoA reductase inhibitor

**Figure 4:**
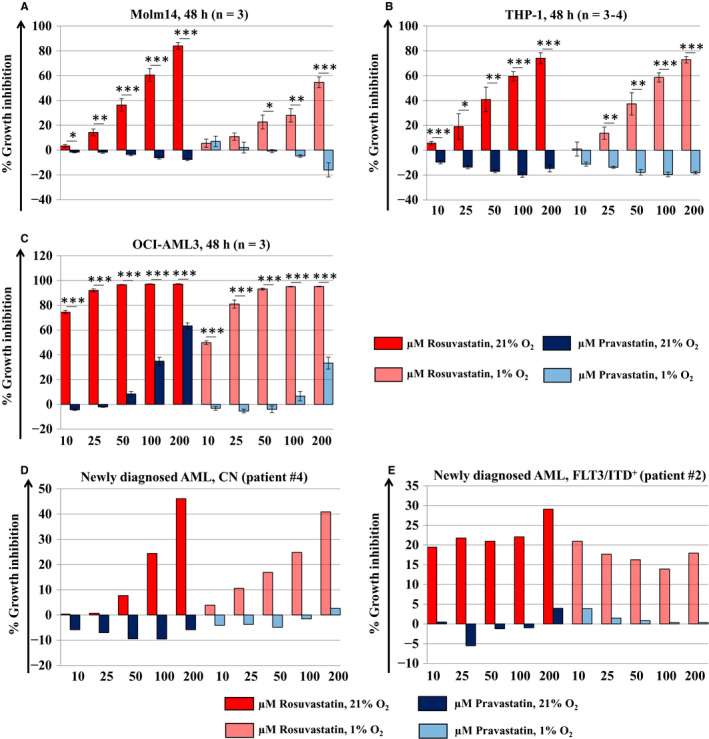
Inhibition of AML cell growth following exposure to rosuvastatin and pravastatin under high and low O_2_ conditions. Rosuvastatin inhibits cell growth in (A) Molm14, (B) THP‐1, (C) OCI‐AML3 as well as in primary AML cells derived from a (D) newly diagnosed AML patient with normal cytogenetics (CN) and a (E) newly diagnosed AML patient harboring a FLT3/ITD mutation. Rosuvastatin‐induced growth inhibition occurred in a dose dependent fashion and was significantly greater compared to pravastatin under both 21% and 1% O_2_ conditions. Pravastatin had mild to no effects in the high and low O_2_ setting. Cells were incubated with 10‐200 μmol/L rosuvastatin or pravastatin at equimolar doses under 21% or 1% O_2_ for 48 h. Growth inhibition was assessed using MTT cell viability assays. The mean ± SEM is based on replicate experiments (n = 3‐4). Statistically significant changes in the percentage of growth inhibition are indicated (*P < .05; **P < .01; ***P < .001)

Applebaum, Penn and colleagues established a proof of concept for mevalonate pathway inhibition using statins as a viable strategy to sensitize AML cells to cytotoxic agents, in addition to providing the first connection between cytarabine treatment and total cholesterol content.^33^, ^34^, ^35^ However, in a clinical setting, while some evidence of benefit was cited, phase I‐II studies of pravastatin in combination with chemotherapy failed to meet the predefined efficacy criteria for success, with the caveat of a relatively low number of subjects.^36^, ^37^ As a result, we utilized rosuvastatin, one of the most potent clinically available HMG‐CoA reductase inhibitor.^38^ We assessed the anti‐leukaemic effects of rosuvastatin and pravastatin against Molm14, THP‐1, OCI‐AML3 and primary AML blasts after 48 hours of drug exposure under high and low O_2_ conditions. In all cells tested and under both oxygenation conditions, rosuvastatin inhibited AML cell growth in a dose‐dependent fashion and was significantly more effective than pravastatin. Pravastatin had mild to no effects at 21% and 1% O_2_ (Figure 4A‐E). Of note, the lacking activity of pravastatin is in line with the observation by Chen et al who reported that pravastatin failed to exert antiproliferative effects against oesophageal and squamous cell carcinoma cells.^39^ Moreover, data published by Menter et al^40^ showed that pravastatin had minimal or no effect on the growth of pancreatic, lung, colon, breast, prostate and bladder cancer cells. The striking difference between pravastatin and rosuvastatin prompted us to confirm that both drugs were actually present in the experimental system in the expected abundance, using standard high‐performance liquid chromatography (Figure S4). The cytotoxic effects of other agents interfering with cholesterol biosynthesis, such as terbinafine, bempedoic and zoledronic acid, against leukaemia cells were comparatively negligible (Figure S5A‐F).

### 3.6 Rosuvastatin potentiates the effects of cytarabine in a diverse set of AML‐related contexts

**Figure 5:**
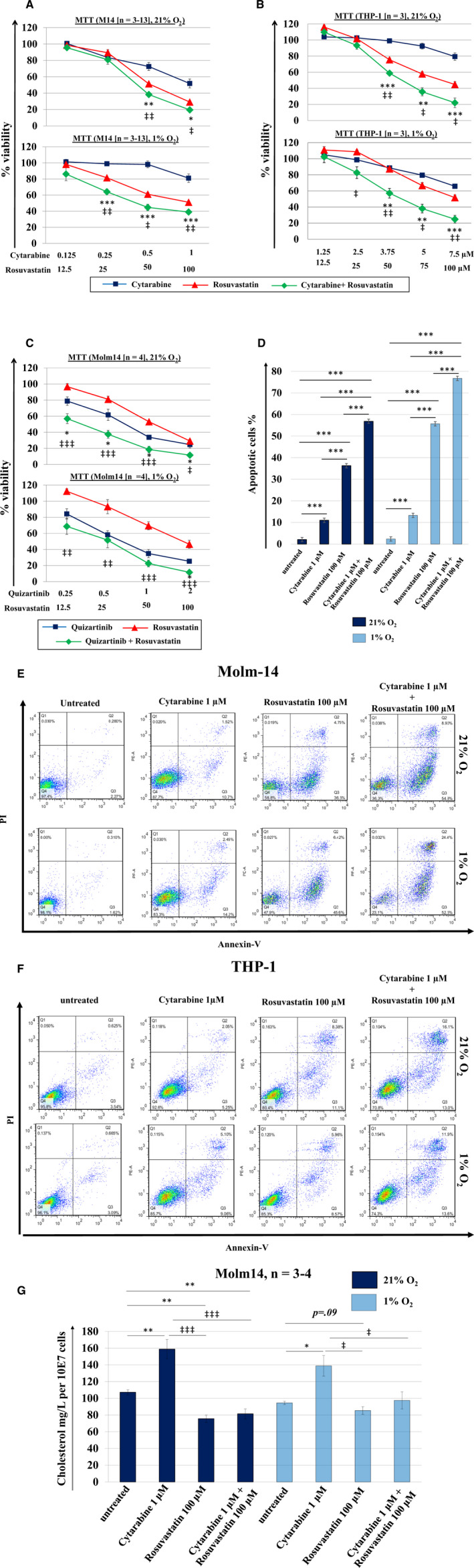
Inhibition of AML cell growth and induction of apoptosis following exposure to cytarabine or quizartinib combined with rosuvastatin under high and low O_2_ conditions. Rosuvastatin significantly enhances cytarabine‐induced growth inhibition of (A) Molm14 and (B) THP‐1 cells under both 21% and 1% O_2_ conditions. (C) Similar results were obtained when rosuvastatin was combined with quizartinib in Molm14 cells. Cells were incubated in drug supplemented medium at the indicated concentrations under 21% or 1% O_2_ for 48 h. Growth inhibition was assessed using MTT cell viability assays. (D) Apoptosis was analyzed by FACS as the percentage of cells positively labeled by Annexin V‐PE. The mean ± SEM is based on replicate experiments (n = 3‐13). Representative data for apoptosis of (E) Molm14 and (F) THP‐1 cells are shown. (G) Rosuvastatin leads to downregulation of intracellular cholesterol levels in Molm14 cells under 21% and 1% O_2_ conditions. While treatment with cytarabine results in increased intracellular cholesterol content, this process is attenuated by the co‐administration of rosuvastatin. Statistically significant changes in the percentage of growth inhibition, apoptotic cells or intracellular cholesterol content are indicated (* or ^‡^ *P* < .05; ** or ^‡‡^ *P* < .01; *** or ^‡‡‡^ *P* < .001)

**Figure 6:**
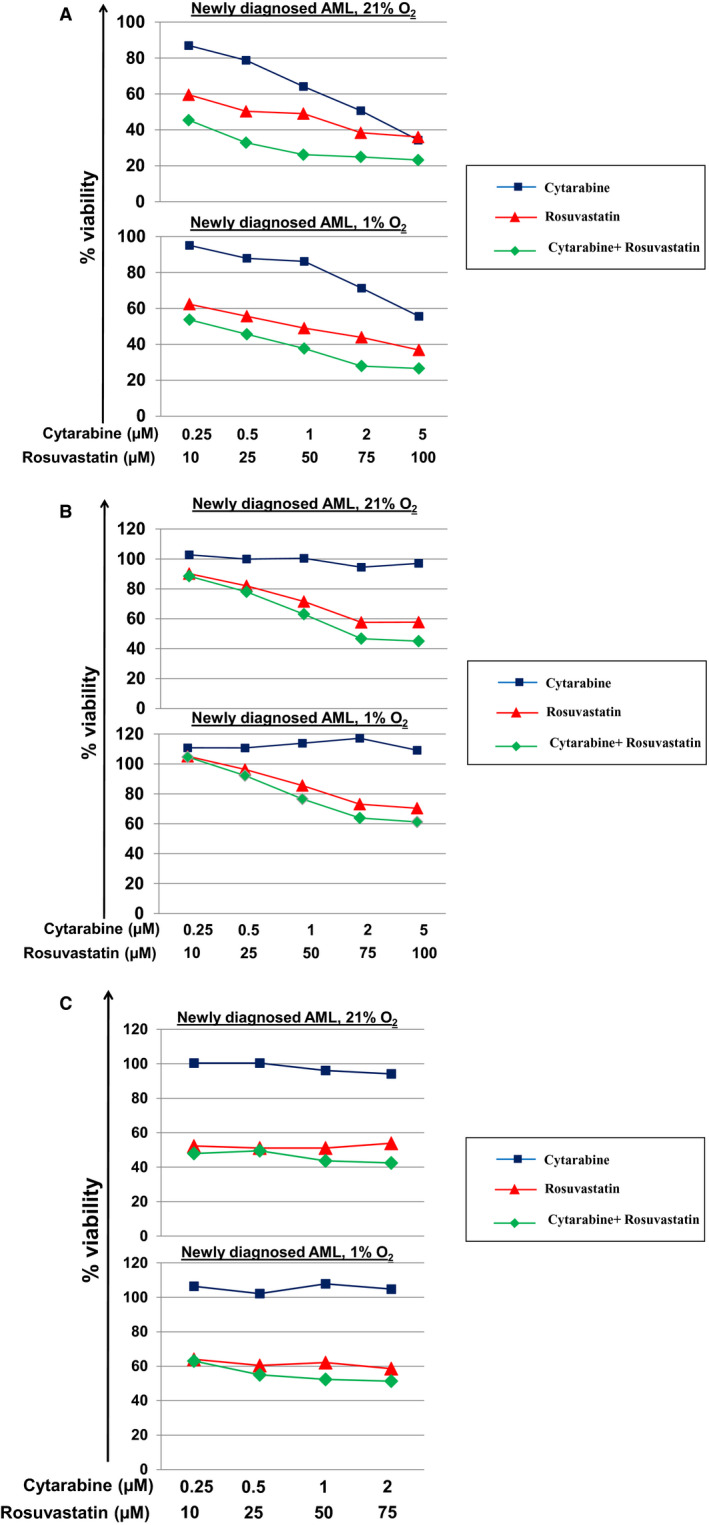
Inhibition of primary AML cell growth following exposure to cytarabine and/or rosuvastatin under high and low O_2_ conditions. Rosuvastatin effectively inhibits growth of primary leukemic blasts derived from (A) a 85 year old, cytogenetically normal AML patient (Patient #4, Table 1), and (B) a 65 year old AML patient harboring a t(9;11) (Patient #5, Table 1), and (C) a 78 year old AML patient harboring a Trisomy 8 (Patient #9, Table 1) whose blasts were resistant to cytarabine. At the doses tested, growth inhibition was greater with rosuvastatin compared to cytarabine under both 21% and 1% O_2_. Combined treatment was more effective in inhibiting primary AML cell growth than single agent therapy with achievement of strong synergistic effects as summarized in Table 2. Cells were incubated in drug supplemented medium at the indicated concentrations under 21% or 1% O_2_ for 48 h. Growth inhibition was assessed using MTT cell viability assays

Next, we tested the validity of the combinatorial approach based on cytarabine and rosuvastatin. Although cytarabine has previously been tested in combination with HMG‐CoA reductase inhibitors in clinical trials,^37^, ^41^ the cytotoxic effects of such combinations yielded only moderate effects. In virtually all systems tested, including conventional AML cell lines (Molm14, THP‐1) and primary cells derived from newly diagnosed AML patients (n = 10) (Figures 5, 6A‐C and Figure S6), the combination was consistently superior to the individual agents. Treatment of Molm14 cells with rosuvastatin revealed potent down‐regulation of intracellular cholesterol levels under 21% (*P *< .01, n = 4) and 1% O_2_ (*P *= .09, n = 3) compared to untreated cells. While treatment with cytarabine significantly increased intracellular cholesterol levels, this response was stalled by co‐treatment with rosuvastatin (Figure 5G). Further, combination indices (CI) suggested additive to synergistic effects in Molm14 (CI_max_ = 0.970 and 0.553, in 21% and 1%, respectively) and TPH‐1 cells (CI_max_ = 0.903 and 0.861, in 21% and 1%, respectively), and synergistic to strong synergistic effects in a diverse panel of primary AML cells (n = 10) as summarized in Table 2. Annexin V/PI staining showed that the combined therapy significantly magnifies the fraction of cells progressing through apoptotic death stages and that the strategy remains effective irrespective of O_2_ abundance (Figure 5D‐F). Of note, rosuvastatin also yielded synergistic effects when combined with quizartinib in Molm 14 cells (CI_max_ = 0.691 [21% O_2_] and 0.706 [1% O_2_]) (Figure 5C).

**Table 2:** Combination index (CI) values for primary AML cells treated with cytarabine combined with rosuvastatin

21% O_2_							
Cell type	n=	Cytarabine (µmol/L)	0.25	0.5	1	2	5
		Rosuvastatin (µmol/L)	10	25	50	75	100
Primary AML (newly diagnosed)	10	Combination index (CI)	0.505^a^	0.254^a^	0.223^a^	0.266^a^	0.416^a^

1% O_2_							
Cell type	n=	Cytarabine (µmol/L)	0.25	0.5	1	2	5
		Rosuvastatin (µmol/L)	10	25	50	75	100
Primary AML (newly diagnosed)	10	Combination index (CI)	0.384^a^	0.452^a^	0.424^a^	0.430^a^	0.529^a^

^a^ Synergy (0 < CI < 0.9).

## 4 DISCUSSION

Decreased O_2_ tension in the neoplastic microenvironment is a well‐established determinant of therapy failure and disease relapse in cancer; however, the experimental evidence remains heavily biased towards solid tumours.^42^, ^43^ In the case of AML, hypoxic niches are naturally present in the bone marrow and are generally thought to act as therapeutic shelters for leukaemic cells.^17^ However, an in‐depth understanding of the underlying processes remains an unmet need. A contributing factor to this knowledge gap is that physiologically relevant oxygenation is only rarely taken into account in AML experimental studies. In the present study, we explored the molecular responses of FMS‐like tyrosine kinase 3/internal tandem duplication–mutated (FLT3/ITD^+^) AML cells to cytarabine and the highly selective FLT3 inhibitor quizartinib under O_2_‐controlled conditions in vitro*.*

Utilizing transcriptomic (RNAseq) and proteomic (reverse phase protein array, RPPA) technologies, our data indicate that the cholesterol biosynthesis pathway is transcriptionally induced under hypoxic stress conditions and attenuated by cytarabine and quizartinib. In contrast, treatment of AML cells with cytarabine led to an increase in total intracellular cholesterol levels irrespective of O_2_ tension. Subsequently, we revisited the concept of cholesterol‐targeted treatment approaches in the management of AML with more potent inhibitors, such as rosuvastatin. We found that rosuvastatin exerts significant anti‐leukaemic activity against a broad selection of AML cells and acts synergistically with cytarabine at different levels of O_2_. Intriguingly, in vitro responses were observed in AML cell lines of different subtypes and in primary cells derived from newly diagnosed AML patients. Our findings firmly support the further investigation of rosuvastatin in combination with classical and investigational therapies for the management of AML.

Our studies provide several new insights regarding the response of cholesterol biosynthesis pathway in the AML context and the derived opportunities for combination therapy. The relevance to cholesterol biosynthesis for AML biology and therapy has been recognized for decades^33^, ^35^; however, to our knowledge this is the first time that this metabolic network is selected in a pathway‐agnostic approach. In retrospect, it appears evident that the low O_2_ experimental component is important, since at 21% O_2_, cholesterol homeostasis drops out of focus, especially in the cytarabine‐treated cells. In contrast to the reproducible increase of intracellular cholesterol levels following cytotoxic therapy, there is strikingly limited information about the response of individual pathway members, particularly under O_2_‐deprived conditions.^33^ To our knowledge, this is the first study revealing a clear contrast between the decreased abundance of cholesterol biosynthesis transcripts and total cholesterol increase in cytarabine‐residual cells. Furthermore, as the RPPA and RNAseq data sets collectively suggest, a similar response appears to involve fatty acid synthesis.

A shift to a scavenger/lipid catabolic programme is the most likely scenario to reconcile this contrast. Consistently, we have confirmed the induction of CD36/FAT, a transmembrane protein that facilitates cholesterol and fatty acid uptake, previously shown to contribute to the resistance of leukaemic cells to therapy.^32^, ^44^, ^45^ Based on its status as canonical SREBF1/2 target, it is not surprising that LDLR features among the robustly down‐regulated transcripts, (Figure S1A), thereby representing an unlikely contributor to the scavenging response, at least in our AML panel. On the other hand, other well‐recognized lipid/cholesterol membrane transporters, including OLR1, SCARF1 and LRP1,^46^ appeared coordinately up‐regulated following drug treatment, with the caveat that these were not individually confirmed.

A partial suppression of lipid anabolism should provide important advantages for at least some types of AML cells. Since de novo cholesterol production is both O_2_‐dependent and uniquely energy‐intensive (36 moles ATP/1 mole final product), this process represents a probable liability under persistent pharmacological and environmental stress. Similar considerations may apply to the closely coordinated de novo fatty acid biosynthesis. In simplistic terms, AML cells with a lower activity window for this programme would be more likely to survive the initial wave of therapy. On the other hand, such a metabolic ‘compromise’ has important limitations since up‐regulation of scavenging transporters should only compensate the deficit of end products but not of biologically active metabolic intermediates (eg terpenoids). This ‘adaptive’ status therefore may also render residual AML cells vulnerable to a pharmacological intervention of the right magnitude at the root of this pathway.

Our results are particularly relevant to the recent clinical studies coordinated by Applebaum and colleagues, testing the benefit of combining conventional cytotoxic agents (cytarabine, idarubicin) with high‐dose pravastatin in newly diagnosed and relapsed/refractory AML. While acceptable toxicity and promising CR/CRp rates of up to 80% and 66% were reported in a phase 1 trial of newly diagnosed and relapsed/refractory AML, respectively, a recent phase 2 trial did not meet the criteria for a positive study.^37^, ^41^ In consonance with this finding, pravastatin has previously been reported to exert only minimal activity against a variety of cancer cells in vitro.^39^, ^40^ While the exact mechanisms underlying this process remain to be elucidated, it has been postulated that the organic anion transporter peptide 1B1 (OATP1B1), which is required for the transmembrane passage of several statins, and to which pravastatin has low affinity, might play a key role in this process.^47^ Of note, a case for combining standard anti‐AML agents with more potent statins was made previously by Penn and colleagues almost two decades ago.^48^, ^49^ However, the promising in vitro effects of cerivastatin were not clinically translatable, due to the withdrawal of this agent from the market.^50^ Rosuvastatin, the statin used in our study, has distinct advantages over other statins in that it carries the highest affinity for HMG‐CoA reductase and is only minimally metabolized by the liver with almost no interaction with CYP 3A4.^38^, ^51^ Our studies extend previous observations by demonstrating that rosuvastatin confers significantly greater anti‐leukaemic activity against AML cells than pravastatin at equimolar doses under both O_2_‐abundant and O_2_‐deprived conditions. To this end, as clinical leukaemias are comprised of heterogeneous populations distributed between the O_2_‐abundant bloodstream and O_2_‐deprived tissue sanctuaries such as the bone marrow, the effectiveness of rosuvastatin alone, or combined with cytarabine, in a diverse mutational and oxygenation context is translationally significant.

In summary, our work demonstrates that the cholesterol biosynthesis signature is affected similarly by cytarabine and quizartinib, two unrelated pharmacological anti‐AML agents, particularly under microenvironmental stress conditions (eg severe hypoxia). We further show that, irrespective of O_2_ tension, rosuvastatin more potently eliminates AML cells compared to pravastatin, which has no effects in the O_2_‐deprived setting and displayed underwhelming activity in AML trials. Our data suggest that the cholesterol biosynthesis programme is amenable to additional translational opportunities within the expanding AML therapeutic landscape and strongly support the further investigation of rosuvastatin‐based combination therapies to enhance targeting residual AML cells that reside in O_2_‐deprived environments, thus addressing an unmet need in AML therapy.

## CONFLICTS OF INTEREST

FC, XW, CN, MG, DP, AR, SR, BG, SG, GAC, HSB and HK do not report any conflicts of interest.

## AUTHOR CONTRIBUTION

FC designed and performed research, collected, analysed and interpreted data. XW designed and performed research, collected and analysed data. CN designed and performed research. MG designed and performed research. DP designed and performed research. AR performed research. SR designed and performed research. BG designed and performed research. SG contributed materials and interpreted data. GAC analysed and interpreted data. HSB contributed materials and interpreted data. HK designed research, analysed and interpreted data, and wrote the manuscript.

## Supporting information

Fig S1‐S6

Table S1A

Table S1B

## Data Availability

The data that support the findings of this study are available from the corresponding author upon reasonable request.
