# Supplementary material for: Classic and targeted anti‐leukaemic agents interfere with the cholesterol biogenesis metagene in acute myeloid leukaemia: Therapeutic implications
Source: J Cell Mol Med. 2020 May 25;24(13):7378–92. doi: 10.1111/jcmm.15339 (PMC7339218; doi:10.1111/jcmm.15339)
Supplement: Supplementary file 1 — Fig S1‐S6 [file JCMM-24-7378-s001.pdf]

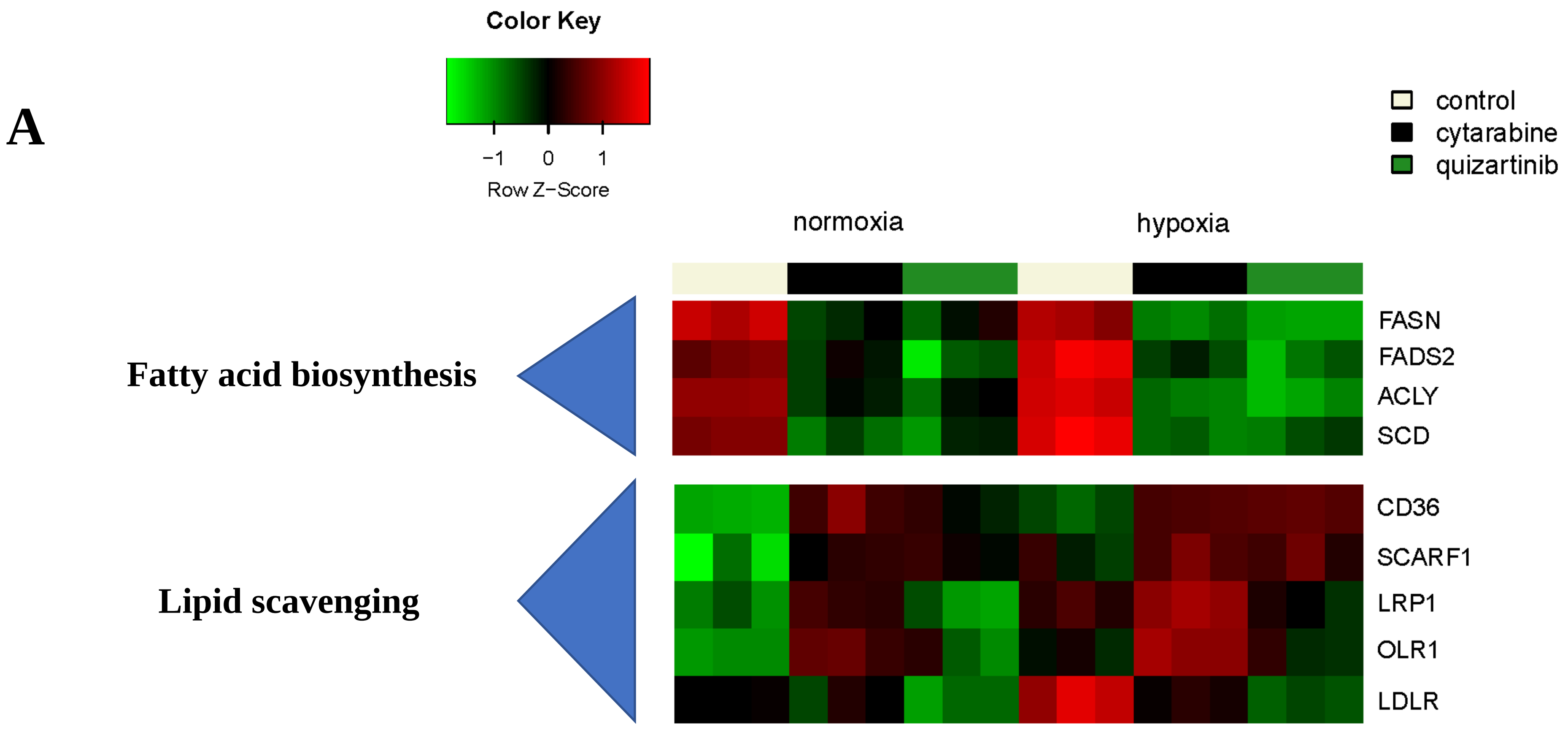

Supplemental Figure 1

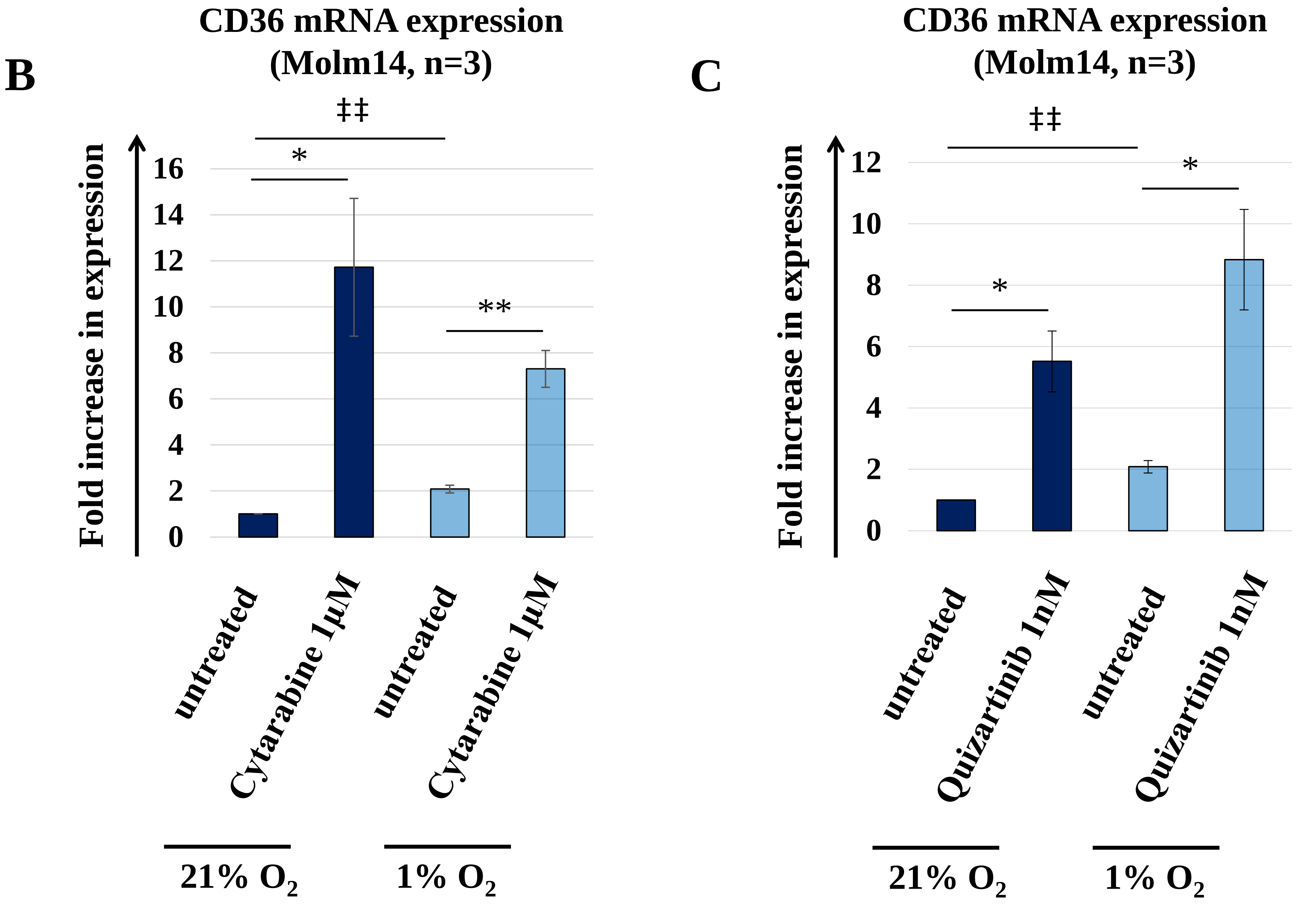

Supplemental Figure 1

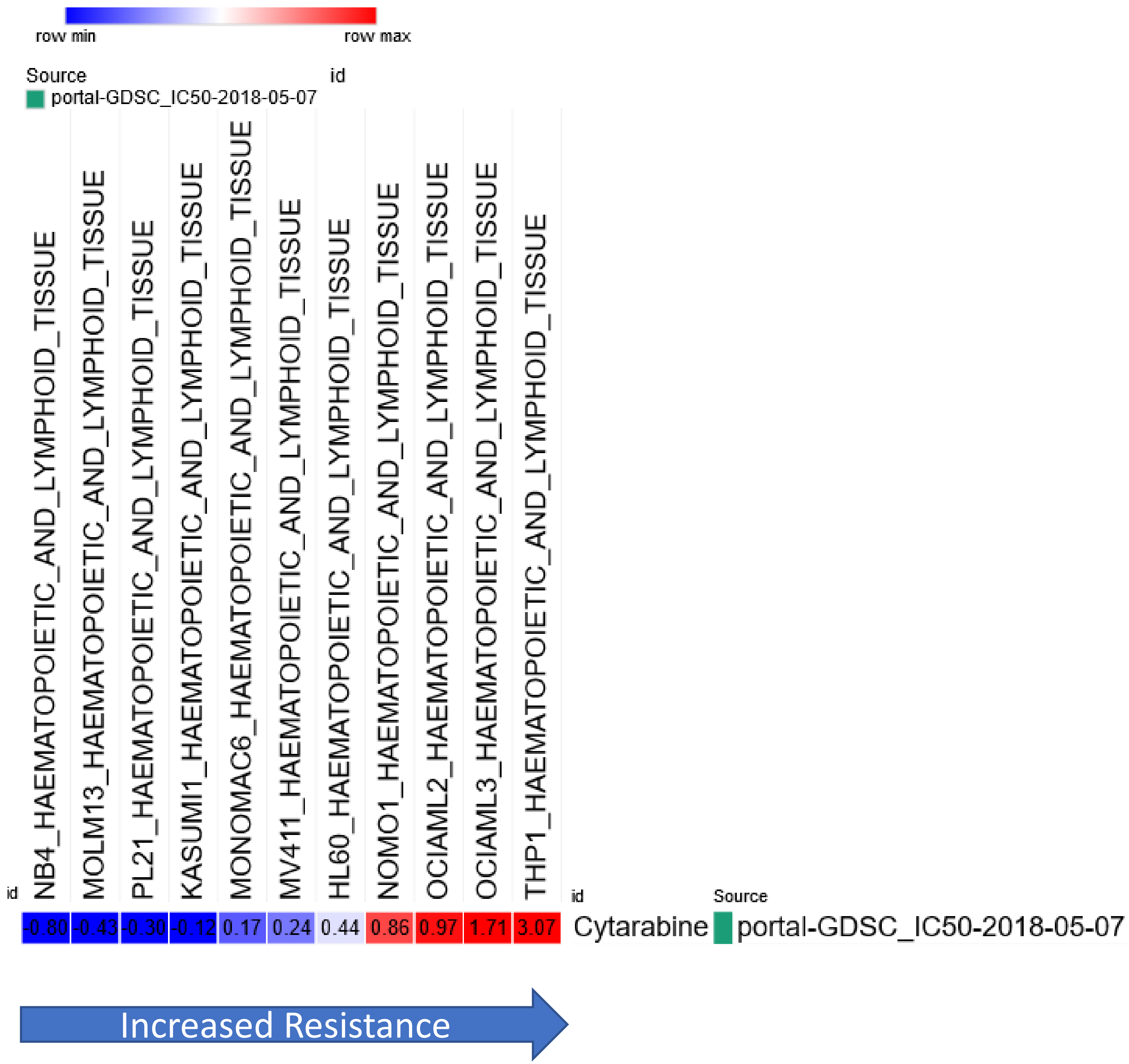

Supplemental Figure 2

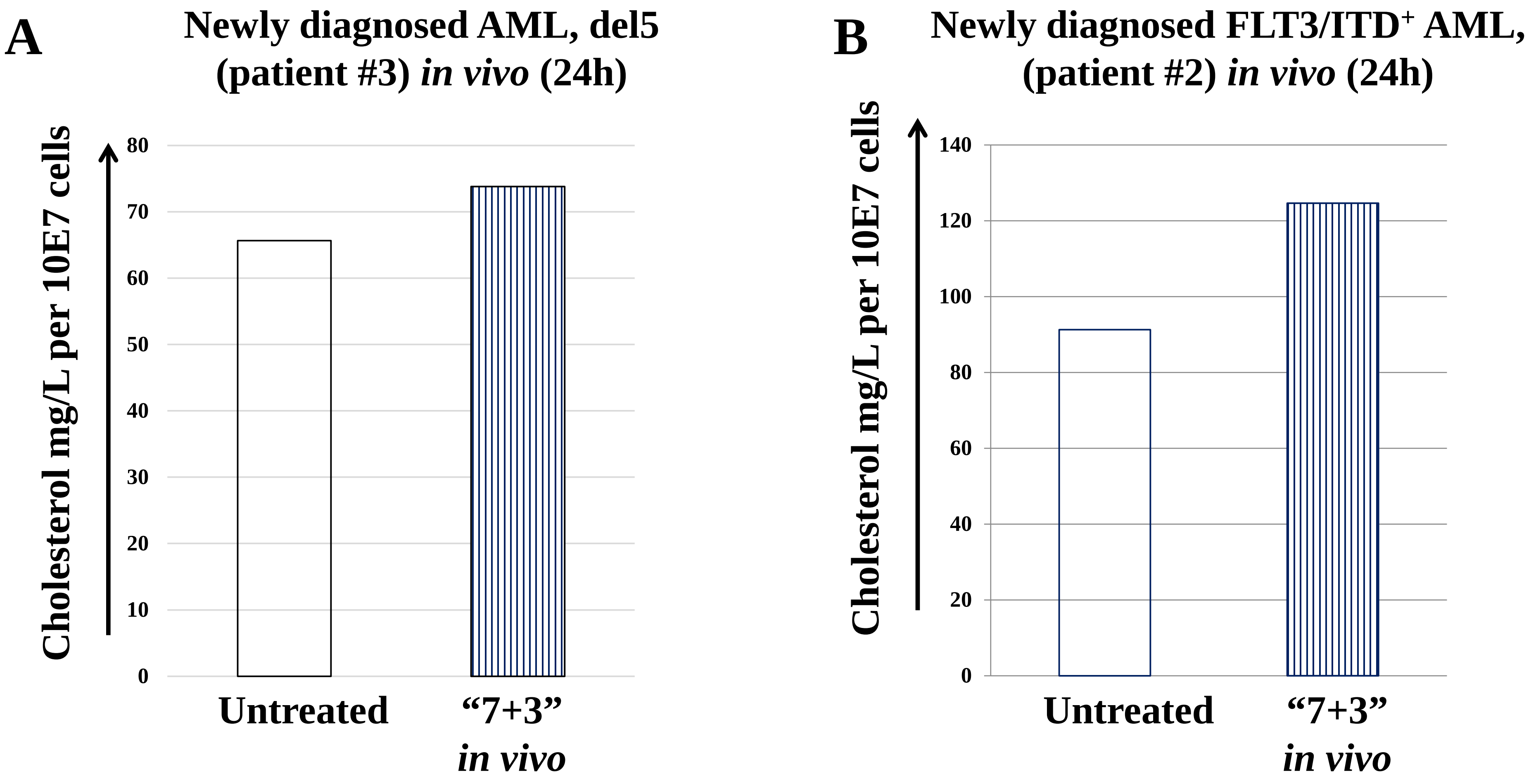

Supplemental Figure 3

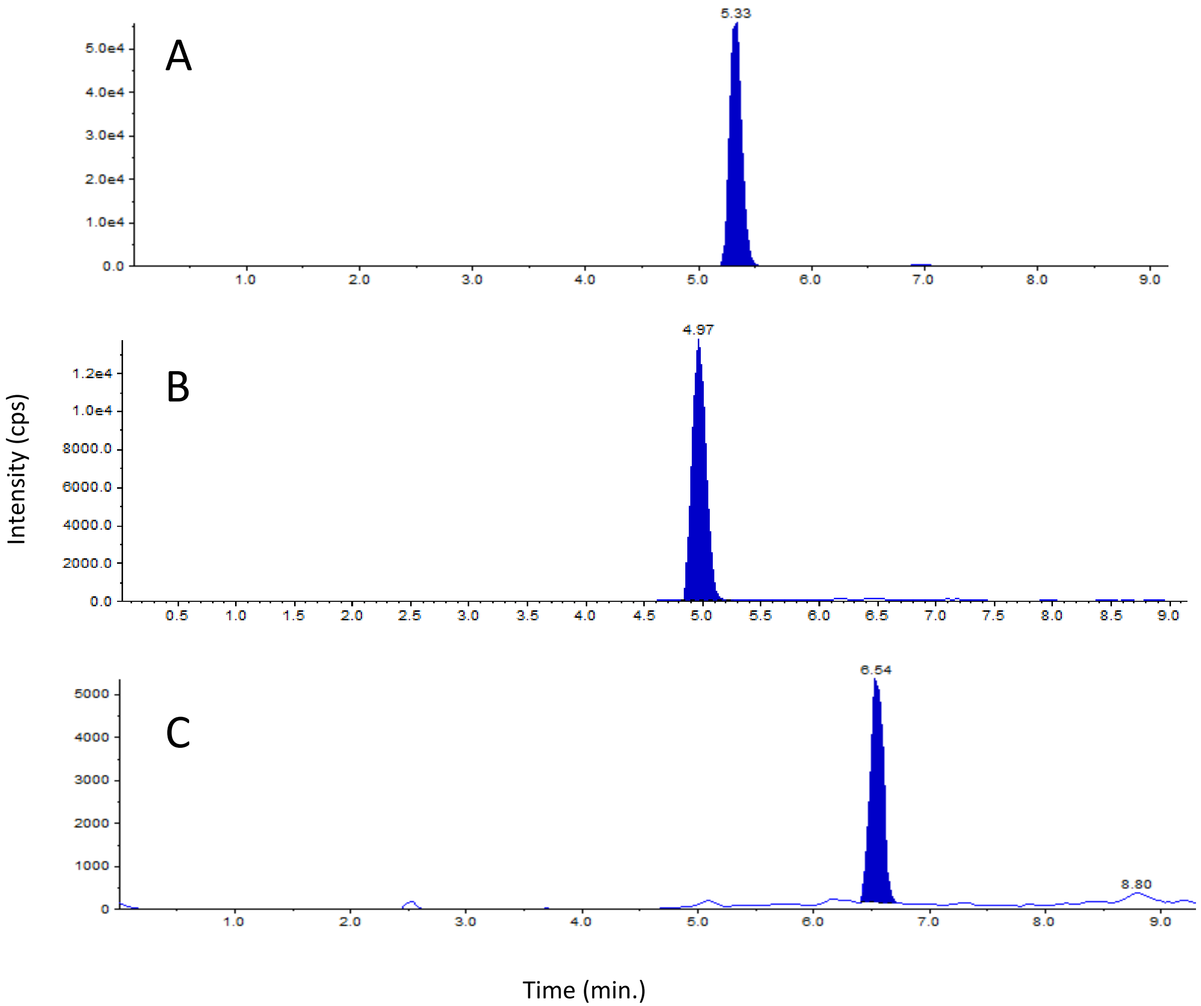

Compound	Q1 (m/z)	Q3 (m/z)	Mode	Declustering Potential (volts)	Entrance Potential (volts)	Collision Energy (volts)	Exit Potential (volts)
Rosuvastatin	482.1	258.2	Positive	110	10	50	15
Pravastatin	423.0	321.2	Negative	-80	-10	-20	-23
Simvastatin	419.4	199.0	Positive	40	10	30	20

Supplemental Figure 4

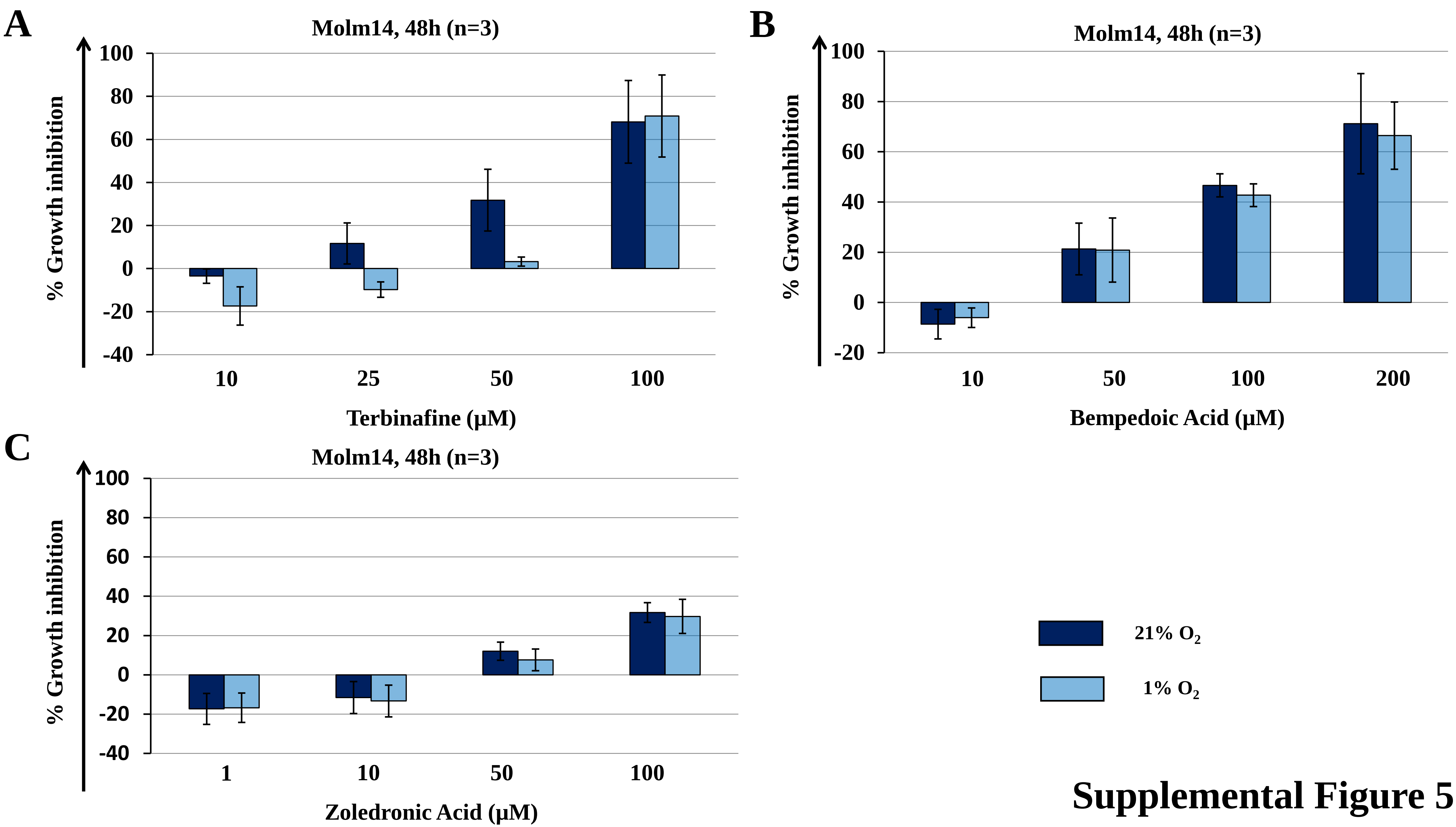

Supplemental Figure 5

D
E
F
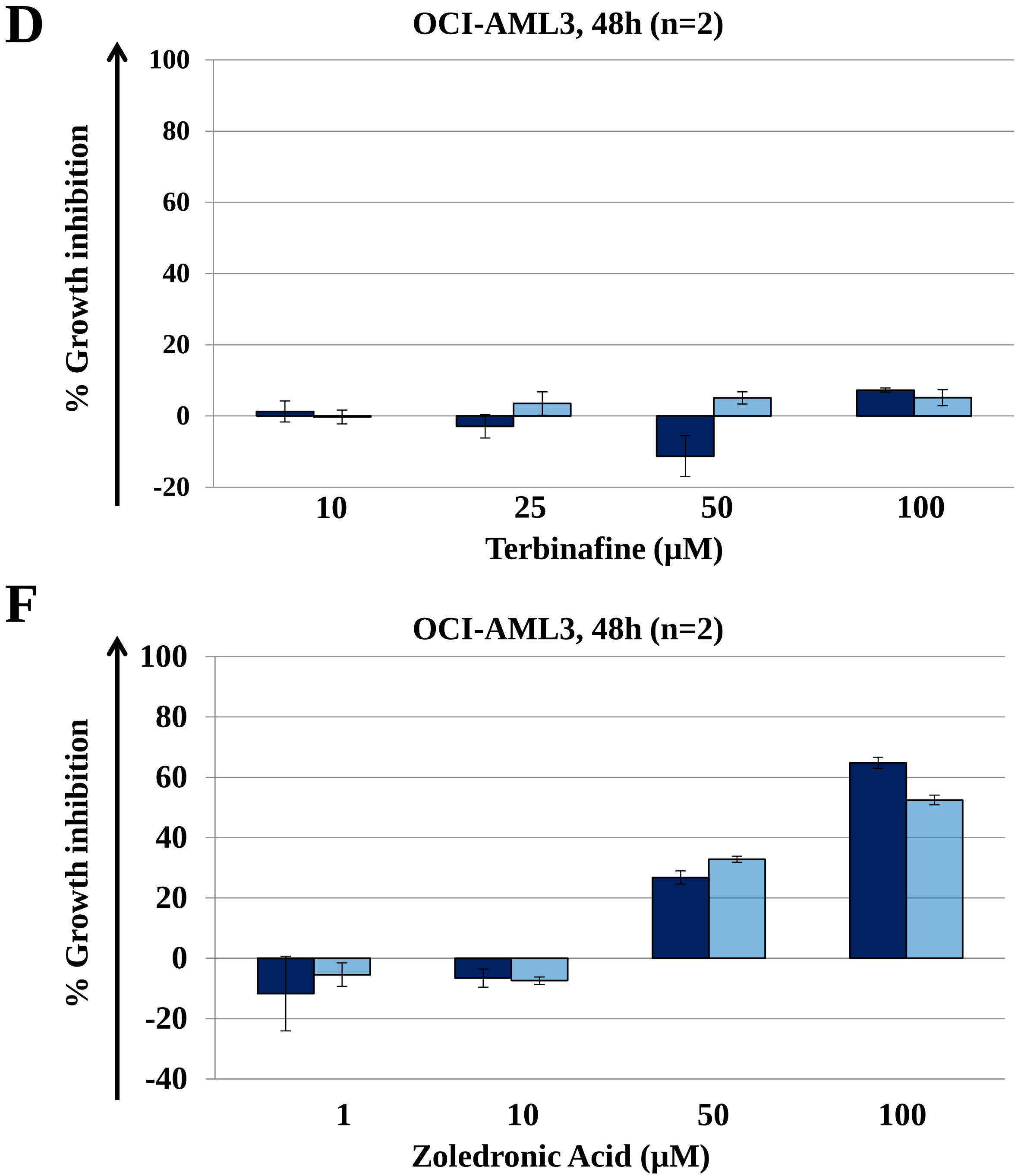

Supplemental Figure 5

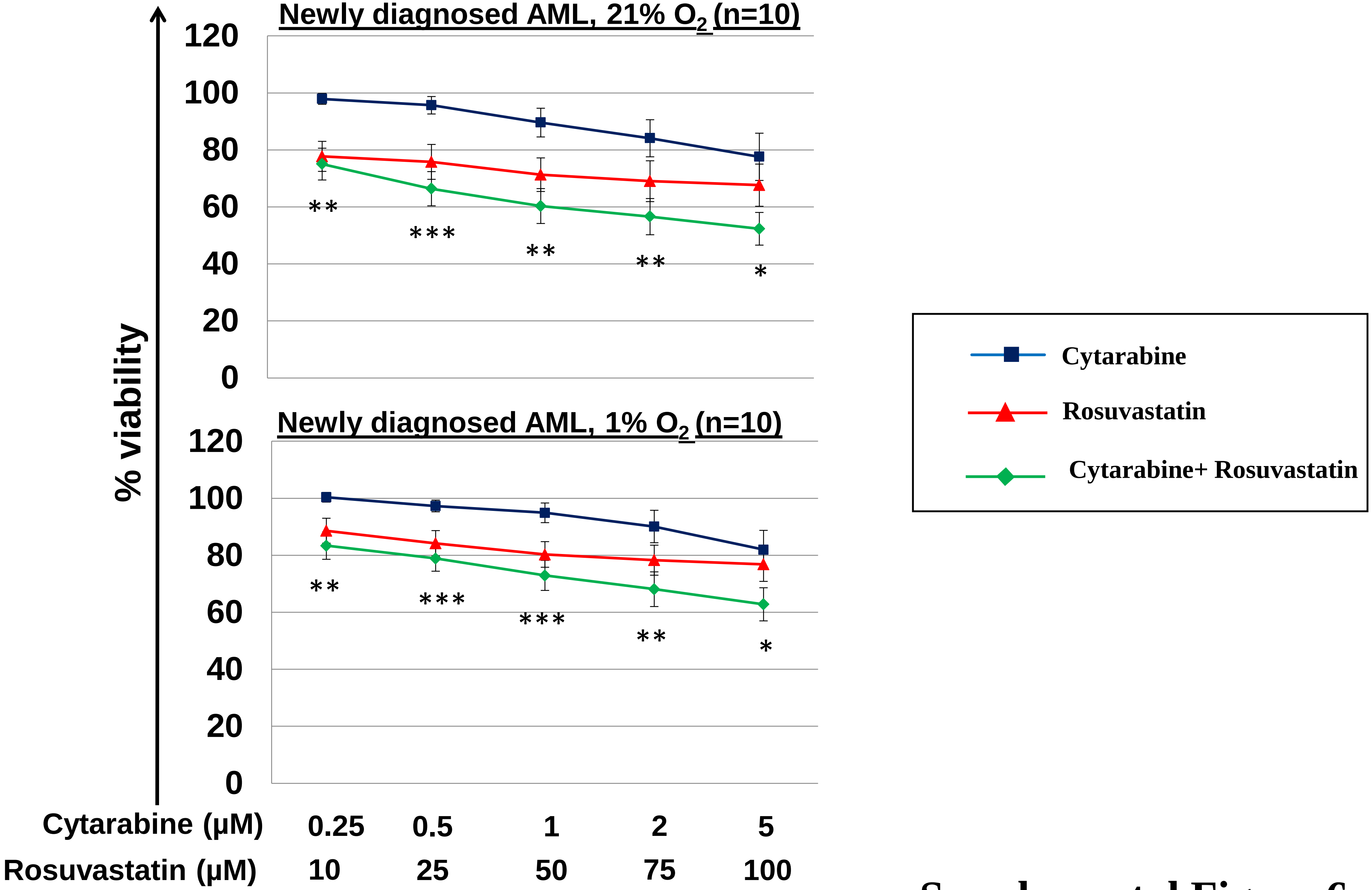

Supplemental Figure 6
